# Heterologous expression of *Mus musculus* immunoresponsive gene 1 (*irg1*) in *Escherichia coli* results in itaconate production

**DOI:** 10.3389/fmicb.2015.00849

**Published:** 2015-08-18

**Authors:** Kiira S. Vuoristo, Astrid E. Mars, Stijn van Loon, Enrico Orsi, Gerrit Eggink, Johan P. M. Sanders, Ruud A. Weusthuis

**Affiliations:** ^1^Bioprocess Engineering, Wageningen University, WageningenNetherlands; ^2^Biobased Products, Wageningen University and Research Centre, WageningenNetherlands

**Keywords:** itaconic acid, *Escherichia coli*, metabolic engineering, *cis*-aconitate decarboxylase, immunoresponsive gene 1, codon optimization, codon harmonization

## Abstract

Itaconic acid, a C5-dicarboxylic acid, is a potential biobased building block for the polymer industry. It is obtained from the citric acid cycle by decarboxylation of *cis*-aconitic acid. This reaction is catalyzed by CadA in the native itaconic acid producer *Aspergillus terreus*. Recently, another enzyme encoded by the mammalian immunoresponsive gene 1 (*irg1*), was found to decarboxylate *cis*-aconitate to itaconate *in vitro*. We show that heterologous expression of *irg1* enabled itaconate production in *Escherichia coli* with production titres up to 560 mg/L.

## Introduction

Itaconic acid is a biotechnologically produced monomer, which can be used as a precursor for many industrially important chemicals such as acrylic plastics, acrylate latices, and absorbents. After being listed as a top 12 value-added biobased chemical compound (Werpy and Petersen, 2004), microbial production of itaconic acid has been of special interest for a decade.

Studies on the biotechnological production of itaconic acid have either focused on strain breeding of natural itaconic acid producers (mainly *Aspergillus terreus*), or on the heterologous expression of *cadA*, the key gene encoding *cis*-aconitate decarboxylase in *A. terreus* in other host organisms (Klement and Büchs, 2013). The economics of itaconic acid production with *A. terreus* are negatively influenced by the slow growth of the organism and the sensitivity of the filamentous pellets to hydro-mechanical stress, which inhibits mass transfer and consequently oxygen supply to the cells. Consequently, the production costs are still too high for industrial application of itaconic acid as a starting material (Steiger et al., 2013). Development of alternative hosts for itaconate production could overcome these problems. *Escherichia coli* may be a suitable candidate host as it grows rapidly under both aerobic and anaerobic conditions and has well-established protocols for genetic modification.

The mammalian immunoresponsive gene 1 (*irg1)* was recently found to have *in vitro cis*-aconitate decarboxylase activity, although it has only 24% amino acid sequence identity with CadA from *A. terreus* (Michelucci et al., 2013). However, both Irg1 and CadA share high identity with MmgE/PrpD family of proteins (Lohkamp et al., 2006; Kanamasa et al., 2008). Irg1 from *Mus musculus* was highly active in mammalian macrophages during inflammation and was linked to having a role in immune defense by catalyzing itaconic acid production (Michelucci et al., 2013), making the enzyme also an interesting candidate for biotechnological itaconic acid production. *E. coli* has been successfully used as a host for itaconic acid production by expressing *cadA* (Li et al., 2011; Okamoto et al., 2014; Vuoristo et al., 2015) and is therefore an interesting candidate to test whether *irg1* can be used for microbial itaconate production.

In earlier studies inclusion body formation was observed in *E. coli* that was overexpressing *cadA* from *A. terreus* (Vuoristo et al., 2015). Expression of heterologous genes in *E. coli* often causes problems such as protein miss-folding and inclusion body formation, which lead to synthesis of inactive enzymes. In recent years many tools, such as codon harmonization, have been developed to optimize a codon usage to its best. Compared to codon optimization, which substitutes codons by the most frequently used codons of the expression host (Makrides, 1996), codon harmonization selects the codons with usage frequencies in the expression host that most closely match the usage frequencies in the native host (Angov et al., 2008). This harmonization leads to better control of the translation speed and may prevent miss-folding of the nascent polypeptide (Angov et al., 2011).

**FIGURE 1:**
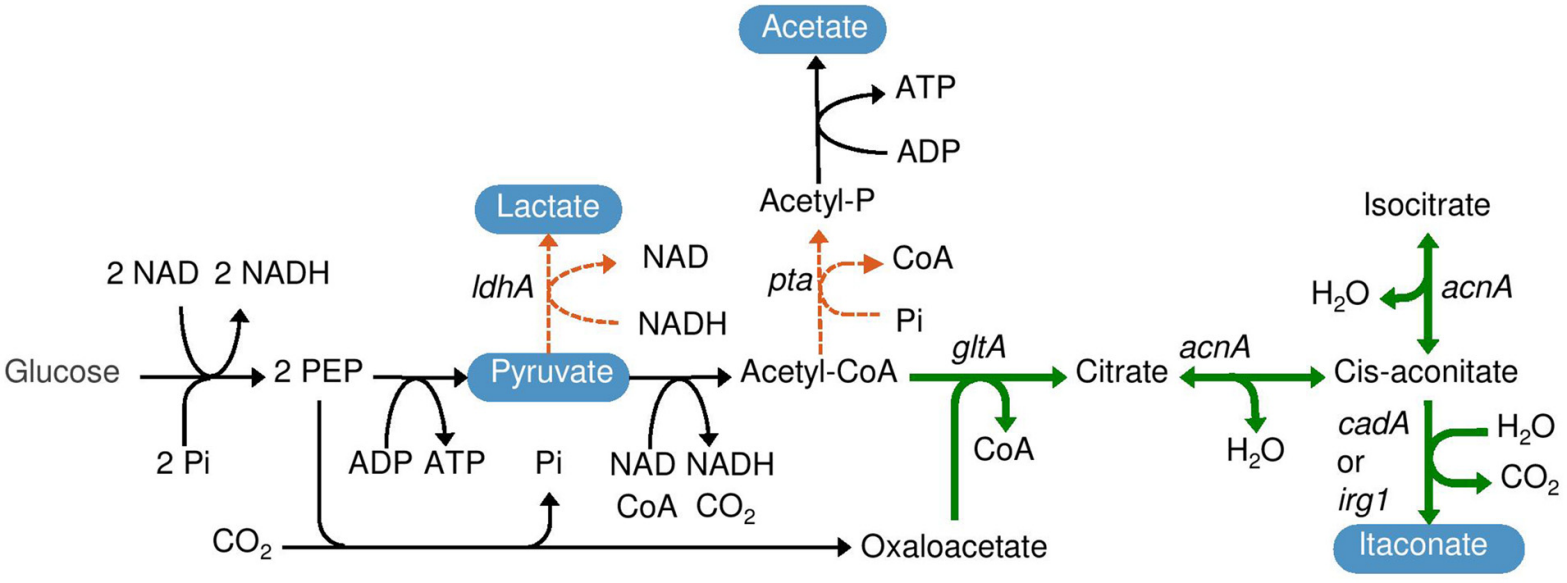
**Itaconate production pathway in *Escherichia coli***. The bold arrows indicate the introduced pathway consisting of genes encoding citrate synthase (*gltA*) and aconitase (*acnA*) from *Corynebacterium glutamicum* and *cis*-aconitate decarboxylase (*cadA)* from *Aspergillus terreus* or immunoresponsive gene 1 (*irg1*) from *Mus musculus*. The dotted lines indicate that phosphate acetyltransferase (*pta*) and lactate dehydrogenase (*ldhA*) were deleted.

In this study, we heterologously expressed *irg1* in *E. coli.* The proposed itaconate pathway in *E. coli* is shown in **Figure 1**. The effect of codon-harmonization on itaconate production and enzyme activity was determined. We showed that expression of mammalian immunoresponsive gene 1 (*irg1*) results in itaconate production in *E. coli*.

## Materials and Methods

### Bacterial Strains and Plasmids

The bacterial strains and plasmids used in this study are listed in **Table 1**.

**Table 1:** *Escherichia coli* strains and plasmids used in this study.

Strains and plasmids	Characteristics	Reference
**Strains**		
BW25113 (DE3)	BW25113 DE3 T7 RNA polymerase	Vuoristo et al. (2015)
BW25113 (DE3) Δ*pta–*Δ*ldhA*	BW25113 Δ*pta*–Δ*ldhA* DE3 T7 RNA polymerase	Vuoristo et al. (2015)
**Plasmids**		
pKV-GAC^opt^	pACYCDuet-1 derivative (Novagen), codon-optimized *gltA* and *acnA* from *Corynebacterium glutamicum*, codon-optimized *cadA* from *Aspergillus terreus*	Vuoristo et al. (2015)
pKV-GA	pACYCDuet-1 derivative, codon-optimized *gltA* and *acnA* from *C. glutamicum*	This study
pKV-GAC^har^	pACYCDuet-1 derivative (Novagen), *gltA* and *acnA* from *C. glutamicum*, codon-harmonized *cadA* from *A. terreus*	This study
pKV-GAI^opt^	pACYCDuet-1 derivative (Novagen), codon-optimized *irg1* from *M. Musculus*, *acnA*, and *gltA* from *C. glutamicum*	This study
pKV-GAI^har^	pACYCDuet-1 derivative (Novagen), codon-harmonized *irg1* from *C. glutamicum*, codon- optimized *acnA*, and *gltA* from *C. glutamicum*	This study

#### Codon-Optimization and Codon-Harmonization

Codon-optimization was done according to the algorithm of OptimumGene^TM^ (GenScript). The codon usage of *cadA* from *A. terreus* and *irg1* from *M. musculus* was harmonized to that of *E. coli.* For this, the codon usage frequency per codon was determined for the native organism and for the host by using the graphical codon usage analyser tool (Fuhrmann et al., 2004). The codons used in the harmonized genes are the *E. coli* codons that mimicked the codon usage frequencies in the native organism the best. Genes were synthesized by GenScript and nucleotide sequences were deposited to NCBI BankIt (accession numbers: *cadA* optimized KM464677, *cadA* harmonized KT273316, *irg1* optimized KT273318, and *irg1* harmonized KT273317).

#### Construction of pACYCDuet-1 Expression Vectors

All strains and plasmids used in this work are given in **Table 1**. All optimized and harmonized *cadA* and *irg1* sequences were expressed by using the T7 promotor in MCS1 of pACYCDuet-1. The control vector pKV-GA was derived from pKV-GAC^opt^ by cloning the *acnA* and *gltA*-containing part of pKV-GAC^opt^ in MCS2 of pACYCDuet-1. In addition, harmonized *cadA*, optimized *irg1* or harmonized *irg1* sequences were cloned in MCS1 of pKV-GA resulting in pKV-GAC^har^, pKV-GAI^opt^ and pKV-GAI^har^, respectively. Constructed plasmids were verified by sequencing.

#### Culture Media

For plasmid construction, *E. coli* strains were cultured at 37°C on lysogeny broth (LB) agar plates or in LB liquid medium and agitation rates of 200 rpm. Medium and plates were supplemented with chloramphenicol (35 μg/mL).

The other cultivations were done in M9 minimal medium (MM), which contained per 1 L: 200 mL 5 × M9 Minimal Salts (BD Difco) supplemented with 50 mM of glucose, 2 mM of MgSO4, 0.1 mM of CaCl_2_, 15 mg of thiamine, and 0.30 mg of selenite and US* trace elements (Panke et al., 1999). Medium was buffered with 0.1 M 3-(*N*-morpholino) propanesulfonic acid (MOPS) and the pH was adjusted to 6.9 with NaOH. Induction of gene expression was started by the addition of 0.5 mM of isopropyl β-D-1-thiogalactopyranoside (IPTG) when the optical density at 600 nm (OD_600_) of the culture reached approximately 0.4.

### SDS-PAGE

For SDS-PAGE, *E. coli* BW25113 (DE3) Δ*pta*–Δ*ldhA* containing either pKV-GA, pKV-GAC^opt^, pKV-GAC^har^, pKV-GAI^opt^, or pKV-GAI^har^ was cultivated for 16 h at 37°C and 200 rpm in 2 mL MM. Subsequently, the overnight cultures were transferred into 20 mL of fresh MM and incubated at 25°C and 200 rpm. When the cultures reached an OD_600_ of approximately 0.4, they were induced with 0.5 mM IPTG and incubated overnight at 25°C and 200 rpm.

Sixteen-milliliter of the cultures were used to make cell free extracts (CFE) according to the Y-PER Yeast Protein Extraction Reagent kit instructions (Thermo Scientific). Four-milliliter of the cultures were harvested by centrifugation (2 min, 14000 rpm), resuspended in a small volume of water, and loaded on pre-casted Criterion XT SDS-PAGE gels (Bio-Rad) together with the CFE’s according to manufacturer’s instructions (Criterion^TM^ Cell, Bio-Rad). The proteins were stained by Bio-Safe Coomassie staining (Bio-Rad). Protein sizes were determined by using Precision Plus Protein^TM^ All Blue Standards ladder (Bio-Rad).

#### Bioreactor Cultures

*Escherichia coli* BW25113 (DE3) Δ*pta*–Δ*ldhA* containing either pKV-GA, pKV-GAC^opt^, pKV-GAC^har^, pKV-GAI^opt^, or pKV-GAI^har^ was cultivated at 25°C in 0.5 L Mini Bioreactors, connected to myControl controller units (Applikon, The Netherlands) with a working volume of 400 ml. The pH was maintained at 6.9 by the automated addition of 2 M NaOH. Bioreactors were inoculated with 5% (v/v) of a pre-culture that was grown at 37°C in a 250 mL Erlenmeyer flasks with 50 mL of MM at 250 rpm for 24 h. The bioreactor cultures were stirred at 500 rpm and sparged with air at 200 mL/min for 8 h, after which the stirring speed was increased to 1200 rpm and the sparging rate was increased to at 400 mL/min. Samples of 2 mL were regularly taken to determine the OD_600_ of the cultures and the concentrations of substrate and products.

#### *cis*-Aconitate Decarboxylase Assay

For enzymatic assays, 30 mL of bioreactor culture was harvested by centrifugation (5 min, 7745 ×*g*) after 17 h of cultivation in the presence of IPTG. CFE were made according to the Y-PER Yeast Protein Extraction Reagent kit instructions (Thermo Scientific). Protein concentrations were determined by using the Total Protein Kit, Micro Lowry, Onishi and Barr Modification (Sigma–Aldrich).

The activity of CadA and Irg1 was measured with *cis*- and *trans*-aconitate according to Vuoristo et al. (2015). Besides, the Irg1 activity was measured by using a method adapted from Michelucci et al. (2013) with the following modifications: CFE’s were incubated with 200 mM of *cis*-aconitate in 25 mM HEPES buffer (pH 7.1) supplied with proteinase inhibitor (cOmplete Protease Inhibitor Cocktail Tablets, Roche) for 50 min at 30°C.

For whole cell assays, 20 mL of bioreactor culture was harvested by centrifugation (5 min, 7745 × *g*) after 17 h of cultivation in the presence of IPTG. Cells were washed with M9 salts in 100 mM MOPS (pH 7.1) and resuspended in 10 ml of the same medium. Twenty-millimeter of substrate (*cis*- or *trans*-aconitate) was added to 2 mL of the cell suspension and incubated at 30°C and 100 rpm. 1 M HCl was added to terminate the reaction, after which the supernatants were analyzed for itaconate formation by HPLC.

#### Analytical Methods

The cell density was determined by measuring the OD_600_ by using a DR6000 spectrophotometer (Hach Lange).

The concentrations of glucose and organic acids were determined by using a Perkin Elmer (200 series) HPLC equipped with an RI detector (PerkinElmer, Flexar) for measuring glucose and an UV detector (PerkinElmer, Flexar UV/V, at 210 nm) for citric acid, itaconic acid, *cis-* and *trans*-aconitic acid. The samples were separated on a Micro Guard Cation H pre-column (30 mm × 4.6 mm, Biorad) and an Aminex HPX-87H column (300 mm × 7.8 mm, Biorad) at 35°C, using 0.5 mL/min of 5 mM H_2_SO_4_ as eluent.

## Results

### Heterologous Expression of *cadA* and *irg1*

Optimized and harmonized gene sequences of *cadA* from *A. terreus* and *irg1* from *M. musculus* were expressed in *E. coli* BW25113 (DE3) Δ*pta*–Δ*ldhA* together with *gltA and acnA* from *Corynebacterium glutamicum*. The strains were cultivated in shake flasks on MM at 25°C and induced with IPTG. Samples of culture pellets and CFE of the cultures were analyzed by SDS-PAGE. A protein band of around 53 kDa appeared on the SDS-PAGE gels, which is the expected size of the proteins encoded by *cadA* and *irg1* (figure provided in the Supplementary Material). The samples of culture pellets gave very thick protein bands at the expected sizes, while much thinner bands were obtained with CFE’s, indicating that CadA and Irg1 were mostly in the form of inclusion bodies. No large differences were observed between the amounts of CadA or Irg1 on the gels after expression of the optimized and harmonized genes. The proteins encoded by *gltA* and *acnA* have sizes of 49 kDA and 114 kDA.

### Enzymatic Assays

*cis*-Aconitate decarboxylase activity was measured in CFE’s of bioreactor cultures after 17 h of cultivation at 25°C in the presence of IPTG. The specific conversion rate of *cis*-aconitate to itaconate in CFE of *E. coli* BW25113 (DE3) Δ*pta*–Δ*ldhA* with pKV-GAC^har^ was 1.39 μmol itaconate/min/mg protein, which was 1.7 times higher than with pKV-GAC^opt^. CadA activities were higher than those previously obtained with cells cultivated at 30 and 37°C (Vuoristo et al., 2015), indicating that inclusion body formation is more suppressed at 25°C. Itaconate formation was not detected with CFE’s of cultures with pKV-GA, pKV-GAI^opt^, or pKV-GAI^har^. Instead, *cis*-aconitate was converted to citrate, which is likely caused by the activity of aconitase in the CFE. Itaconate formation was also absent in cell lysates of pKV-GAI^opt^ and pKV-GAI^har^ that were not centrifuged, indicating that Irg1 activity is not present in the membrane fraction of the cells. *Trans*-aconitate was not converted by any of the CFE’s.

Irg1 activities were also determined according to Michelucci et al. (2013). Again, no itaconate formation was detected under these conditions (results not shown). The absence of conversion of *cis-*aconitate to itaconate in CFE of *E. coli* BW25113 (DE3) Δ*pta*–Δ*ldhA* expressing *irg1* suggests that Irg1 was inactivated in the period between the harvest of the cells and the measurements.

### Whole Cells Assays

**FIGURE 2:**
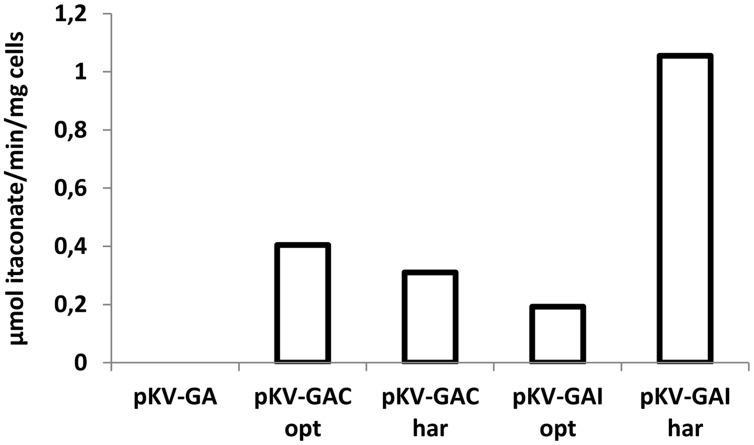
**Conversion of *cis*-aconitate to itaconate (μmol itaconate/min/mg cells) by whole cells of *E. coli* BW25113 (DE3) Δ*pta*–Δ*ldhA* containing pKV-GA, pKV-GAC^opt^, pKV-GAC^har^, pKV-GAI^opt^, and pKV-GAI^har^.** The average values of duplicate measurements are given.

Conversion of *cis*-aconitate to itaconate by cells of *E. coli* BW25113 (DE3) Δ*pta*–Δ*ldhA* containing pKV-GA, pKV-GAC^opt^, pKV-GAC^har^, pKV-GAI^opt^, and pKV-GAI^har^ was measured by incubating cells in the presence of 20 mM of *cis*- or *trans*-aconitate and measuring the formation of itaconate in the supernatant. Whole cells containing pKV-GAC^opt^, pKV-GAC^har^, pKV-GAI^opt^, and pKV-GAI^har^ converted *cis*-aconitate to itaconate. *Trans*-aconitate was not converted (data not shown). The highest conversion rate was observed with pKV-GAI^har^ (1.10 μmol itaconate/min/mg cells, **Figure 2**), which was much higher than with pKV-GAI^opt^ (0.19 μmol itaconate/min/mg cells). The conversion rates of pKV-GAC^opt^ and pKV-GAC^har^ were (0.41 and 0.31 μmol itaconate/min/mg cells).

### Bioreactor Cultures

**FIGURE 3:**
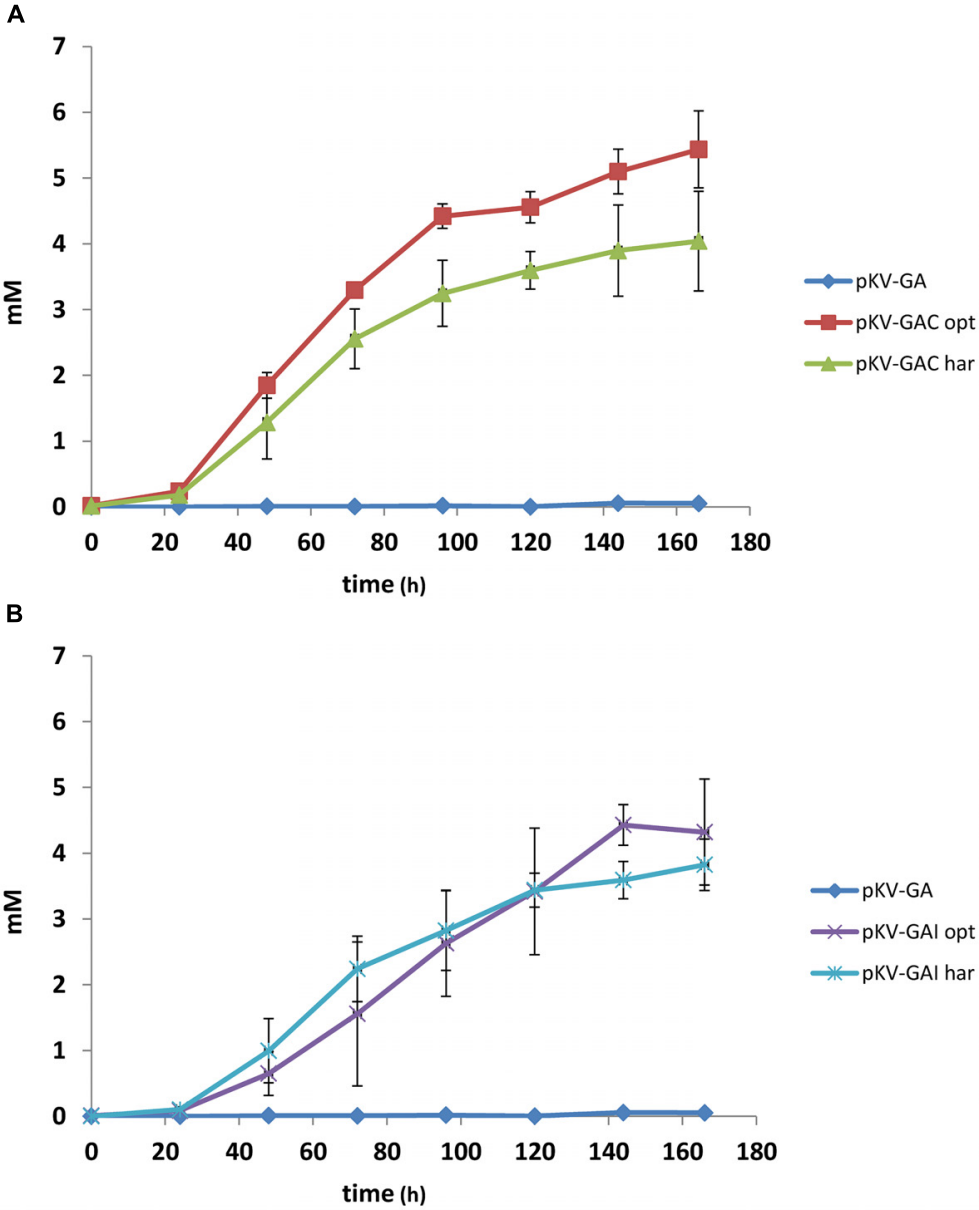
**Itaconate production in bioreactor cultures containing *E. coli* BW25113 (DE3) Δ*pta*–Δ*ldhA* with pKV-GA, pKV-GAC^opt^, or pKV-GAC^har^**(A)** or pKV-GA, pKV-GAI^opt^, or pKV-GAI^har^ (B).** The average values and SD of duplicate cultures are given.

The effect of expression of *cadA* and *irg1* on itaconate production was studied in *E. coli* BW25113 (DE3) Δ*pta*–Δ*ldhA*, in which the genes encoding phosphate acetyltransferase and lactate dehydrogenase were disrupted. *gltA* and *acnA* from *C. glutamicum* were expressed together with the target genes. The strains were cultivated under aerobic conditions in pH-controlled bioreactors on MM at 25°C. Itaconate was produced in all cultures, except in the control pKV-GA (**Figure 3**). Strains containing the harmonized genes (pKV-GAC^har^ and pKV-GAI^har^) produced slightly less itaconate than those containing the optimized genes (pKV-GAC^opt^ and pKV-GAI^opt^). The latter strains produced 5.5 and 4.3 mM, respectively.

## Discussion

Since the identification of the gene encoding *cis*-aconitate decarboxylase (*cadA*) in *A. terreus* (Kanamasa et al., 2008), there has been a growing interest to produce itaconic acid in recombinant hosts such as *E. coli* (Liao and Chang, 2010; Li et al., 2011). Metabolic engineering strategies to increase the flux through the citric acid cycle and to reduce by-product formation have significantly increased the itaconate titres obtained with *E. coli* (Okamoto et al., 2014; Vuoristo et al., 2015), but they are still far below those obtained with *A. terreus* (Hevekerl et al., 2014). One of the possible bottlenecks in *E. coli* is the low activity of the heterologous CadA.

Although *E. coli* is a widely used cell-factory for the production of proteins and chemicals, problems such as inclusion body formation and low enzyme activity are often associated with heterologous production of proteins. For instance, low enzyme activities may occur when the frequency of synonymous codons in foreign coding DNA significantly differs from that of the host (Rosano and Ceccarelli, 2014). Harmonization of the codon usage frequencies of the target gene with those of the expression host can increase their expression (Angov et al., 2008; Van Zyl et al., 2014). We compared expression of codon optimized and harmonized sequences of *cadA* and *irg1* by determining enzyme activities and performance in bioreactor cultures. The specific enzymatic activity with harmonized *cadA* was 1.7 times higher than with optimized *cadA*. In contrast, the specific conversion rate of *cis*-aconitate by whole cells containing pKV-GAC^har^ was only 75% of the rate observed with pKV-GAC^opt^, and the titre obtained in bioreactor cultures with pKV-GAC^har^ was 74% of that obtained with pKV-GAC^opt^. This suggests that other factors, like itaconate export, by-product formation, or levels of other enzymes involved in itaconate biosynthesis have a larger effect on itaconate production than the activity of CadA.

Okamoto et al. (2014) measured increased intracellular itaconate concentrations in their production strains and suggested that extracellular secretion of itaconate in *E*. *coli* limits the production. Itaconate transport has been studied in *Aspergillus* species and a few putative candidates have been characterized (Li et al., 2011). Expression of *A. terreus* mitochondrial transporter *mttA* and plasma membrane transporter *mfsA* were beneficial to production in *A. niger* (Li et al., 2013; van der Straat et al., 2014). So far, itaconate transporters have not been identified in *E. coli*, and the transport mechanism is unknown, which makes them obvious research targets for the future.

Besides CadA, alternative *cis*-aconitate decarboxylases can facilitate itaconate production. Recently, the gene product of immunoresponse gene 1 (*irg1*) from *M. musculus* was shown to catalyze the decarboxylation of *cis*-aconitate to itaconic acid *in vitro* (Michelucci et al., 2013). We were unable to detect any *cis*-aconitate decarboxylase activity in CFE’s of *E. coli* cultures expressing *irg1*, although the cultures were producing itaconate. This indicates that the enzyme was inactivated during preparation of CFE’s. Whole cells of pKV-GAI^har^ converted *cis-*aconitate nearly 5.5 times as fast as pKV-GAI^opt^. Still, the titre of pKV-GAI^har^ was less than the titre of pKV-GAI^opt^.

We showed for the first time that introduction of this mammalian enzyme also results in itaconate production in *E. coli* with titres up to 560 mg/L. The titres are similar to those obtained with *cadA*-expressing *E. coli* strains (700 mg/L), although the protein sequences are only 24% identical.

## Conclusion

We have successfully expressed a mammalian *cis*-aconitate decarboxylase encoded by *irg1* from *M. musculus* in *E. coli.* Titres up to 560 mg/L of itaconate were obtained with *irg1*, which are comparable to those obtained with *cadA* from *A. terreus* (700 mg/L). Codon harmonization increased the activity CadA in CFE, but this did not result in higher itaconate production in bioreactor cultures, suggesting that other factors are limiting. Besides *irg1* from mouse, also other immunoresponse genes are known. Many of them may possess CadA activity and it should be investigated which ones are best for itaconate production.

## Author Contributions

KV performed the bioreactor experiments, enzymatic assays and drafted the manuscript. AM participated in the design of the experiments and helped to draft the manuscript. SL and EO constructed the strains and verified the expression of heterologous genes on SDS-PAGE. GE supervised the project and commented the manuscript. JS conceived the project and commented the manuscript. RW conceived, designed and supervised the project, and contributed to the writing of the manuscript. All authors read and approved the final manuscript.

## Conflict of Interest Statement

The authors declare that the research was conducted in the absence of any commercial or financial relationships that could be construed as a potential conflict of interest.
