# Supplementary material for: Heterologous expression of Mus musculus immunoresponsive gene 1 (irg1) in Escherichia coli results in itaconate production
Source: Front Microbiol. 2015 Aug 18;6:849. doi: 10.3389/fmicb.2015.00849 (PMC4539527; doi:10.3389/fmicb.2015.00849)
Supplement: Supplementary file 1 [file Data_Sheet_1.DOCX]

**Supplementary material**

L control CadA^opt^ CadA^opt^ CadA^har^ Irg1^opt^ Irg1^har^ L

P C P C P C P C P C P C

P C


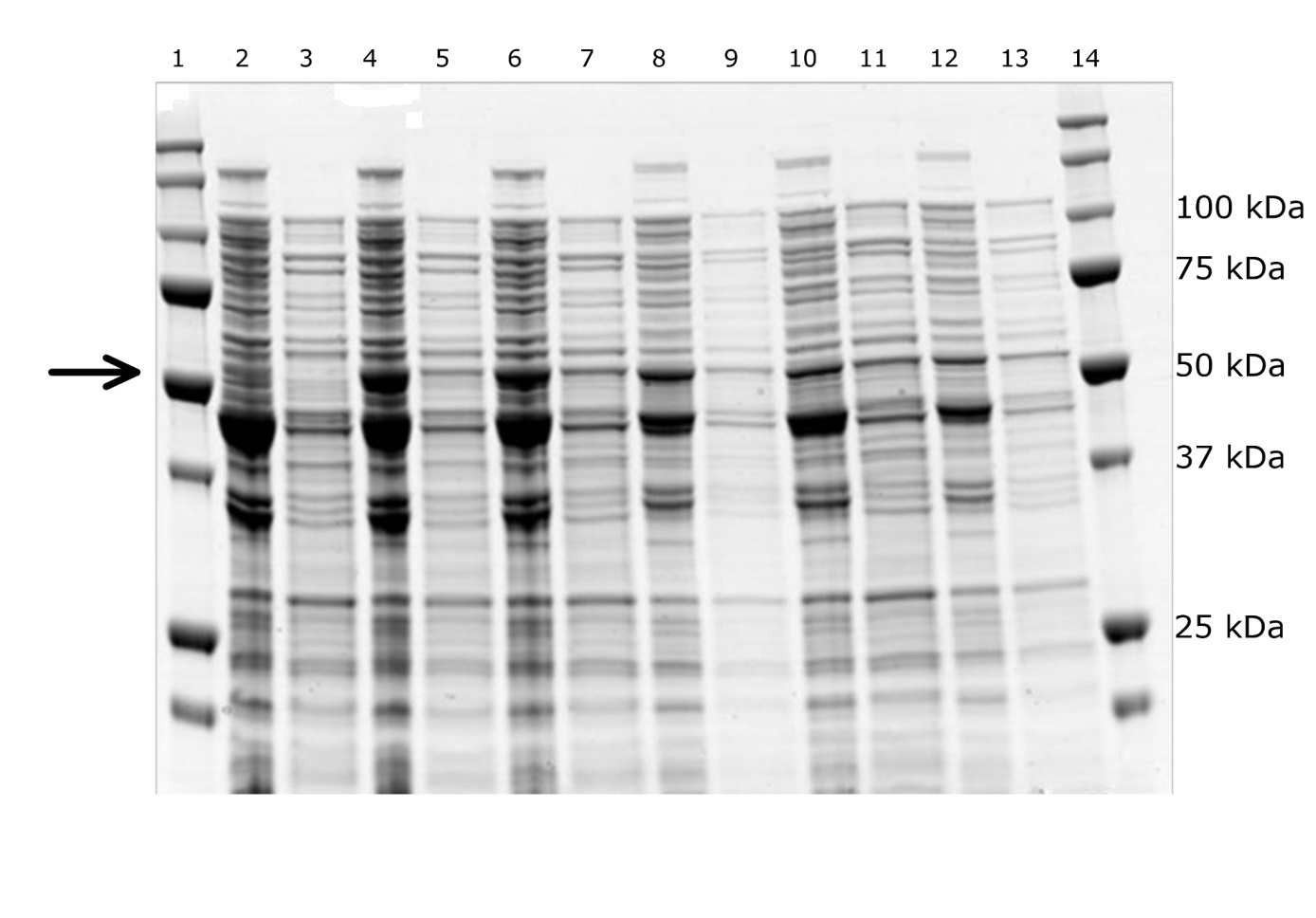
Protein profiles of *E. coli* BW25113 (DE3) Δ*pta*-Δ*ldhA* pKV-GA, pKV-GAC^opt^, pKV-GAC^har^, pKV-GAI^opt^ and pKV-GAI^har^. Lane 1 and 14: Precision Plus Protein All Blue standards. Lane 2-3: pKV-GA, 4-7: pKV-GAC^opt^, 8-9:pKV-GAC^har^, 10-11:pKV-GAI^opt^, 12-13:pKV-GAI^har^. Even lanes contain culture pellets (P), odd lanes contain CFE’s (C). The arrow indicates the position of CadA and Irg1 around 53 kDa.
