# Supplementary material for: Heterologous expression of Mus musculus immunoresponsive gene 1 (irg1) in Escherichia coli results in itaconate production
Source: Front Microbiol. 2015 Aug 18;6:849. doi: 10.3389/fmicb.2015.00849 (PMC4539527; doi:10.3389/fmicb.2015.00849)

Supplementary material

HPLC compound list and retention times

Component	Retention time	Channel
cis-aconitate	9.194	UV
citric acid	9.957	UV
glucose	10.984	RI
pyruvic acid	11.688	UV
trans-aconitate	12.608	UV
succinic acid	14.575	RI
lactic acid	15.363	RI
itaconic acid	16.188	UV
formic acid	17.161	RI
acetic acid	18.747	UV
**propionic acid_int.std**	**22.126**	UV
MOPS	23.193	RI
ethanol	25.961	RI

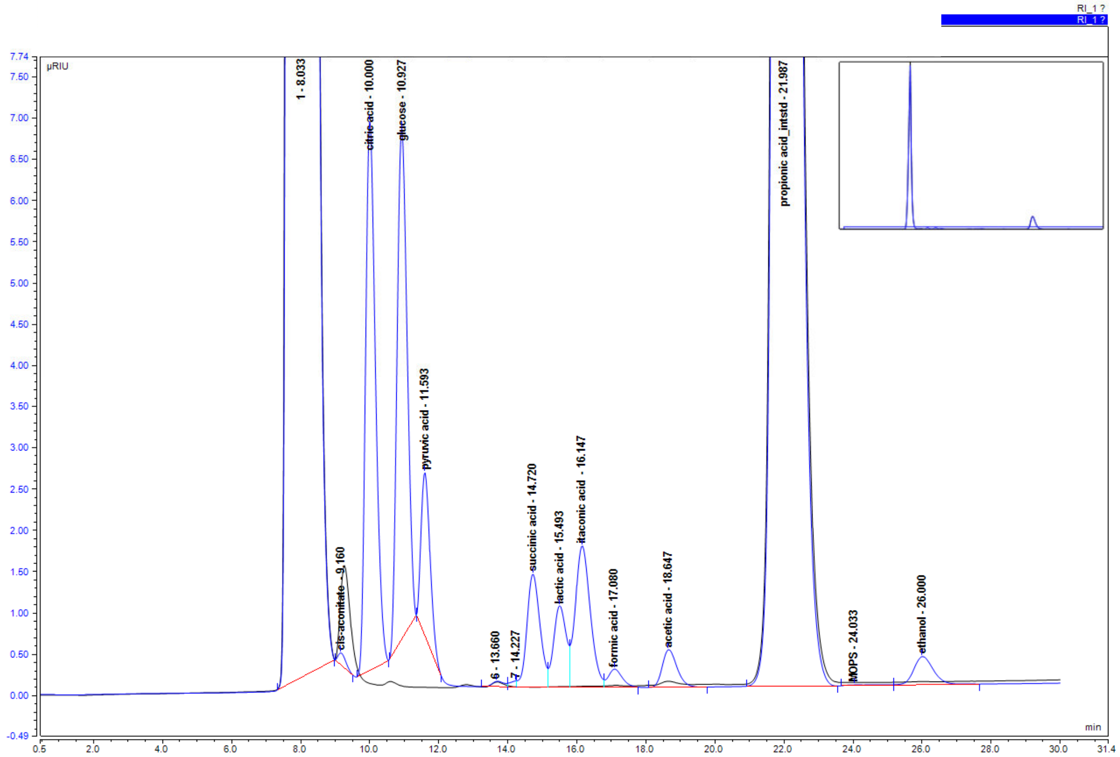

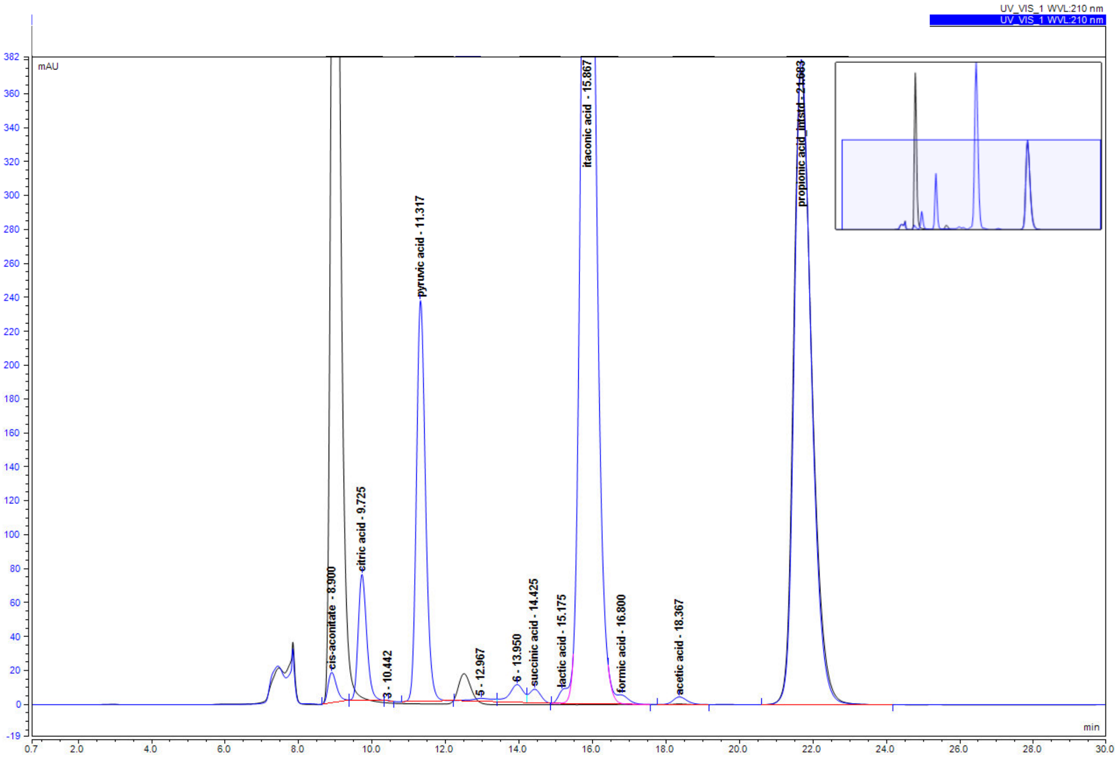

Supplement: Supplementary file 2 [file Data_Sheet_2.PDF]
